# Herpesviruses and autophagy: An intracellular conflict

**DOI:** 10.1080/21505594.2026.2697089

**Published:** 2026-07-14

**Authors:** Qinqin Sun, Lianshun Zhang, Junze Chen, Qiang Fu

**Affiliations:** a School of Animal Science and Technology, Foshan University, Foshan, China; b Foshan University Veterinary Teaching Hospital, Foshan University, Foshan, China

**Keywords:** Herpesvirus, autophagy, replication, infection

## Abstract

Herpesviruses are a class of viruses capable of causing various diseases in humans and animals. These pathogens have the ability to remain latent within the host cell for extended periods, often remaining dormant until immune function is compromised. Extensive research indicates that autophagy, as a key intracellular defense mechanism, is closely associated with the herpesvirus life cycle. Following cellular invasion, herpesviruses evade immunity and sustain infection by modulating autophagy formation, blocking autophagy-lysosome fusion, or selectively degrading key immune molecules via autophagy. Concurrently, autophagy exerts antiviral effects under specific conditions by restricting viral replication through receptor-mediated degradation or non-canonical pathways. This review summarizes recent advances in herpesvirus-autophagy interactions, outlines the commonalities and unique features of viral autophagy regulation, and explores its impact on viral life cycles and host immune responses. The theoretical foundations for identifying novel antiviral targets and developing precision interventions for herpesvirus-related diseases are provided.

Herpesviruses are a category of double-stranded deoxyribonucleic acid viruses classified into three distinct subfamilies. Notable herpesviruses within the α subfamily include HSV-1/2, MDV, VZV, PRV, FHV and BoHV-1. Common herpesviruses of the γ subfamily include EBV and KSHV.

Herpesviruses are highly diverse and evolutionarily advanced viruses that have developed a variety of strategies to utilize autophagy. These strategies include the formation of autophagosomes and the subsequent degradation of their contents by lysosomes. The virus can manipulate these processes depending on the specific type of virus, its lifecycle, and the host cells it infects. Notably, viruses employ diverse tactics to disrupt autophagy, thus evading their elimination and ensuring their persistent presence within the infected host. However, in some cases, viruses may also exploit the process of autophagy by inducing the formation of autophagosomes and utilizing the intracellular transport of vesicles during replication to complete their lifecycle. Upon detection of a herpesvirus stimulus, the cell initiates a series of measures to counter viral intrusion, including the activation of autophagy to combat the virus or to enable the host to control viral infection through the innate immune response [1]. For example, in classical autophagy, certain herpesviruses encode viral Bcl-2 homologs (vBcl-2) that inhibit Beclin1 [2–5] while Kaposi’s sarcoma-associated herpesvirus (KSHV) targets vacuolar protein sorting 34 (Vps34) [6]. MCMV targets vacuolar protein sorting 26 (Vps26), thereby modulating autophagy regulation [7]. In addition, autophagy-related protein 3 (Atg3) is inhibited by the FLICE-like inhibitory protein (FLIP), and KSHV exploits this pathway by encoding a viral FLIP homolog (vFLIP), which suppresses Atg3 activity and consequently impairs microtubule-associated protein 1 light chain 3 (LC, an Atg8 homolog) lipidation [8]. Certain viruses can also inhibit autophagy by impeding autophagy-lysosome formation. The P protein of human parainfluenza virus type 3 (HPIV3) has been shown to impede the fusion of autophagosomes and lysosomal compartments, thus increasing viral replication [9]. This mechanism has also been observed in varicella-zoster virus (VZV) [10], further supporting its role in viral pathogenesis. In selective autophagy, cellular autophagy can function as a natural defense mechanism against viral invasion and replication during infection. In this context, autophagy receptor proteins, such as sequestosome 1 (p62, SQSTM1), next to BRCA1 gene 1 protein (NBR1), and optineurin (OPTN), play crucial roles. These proteins can recognize and bind ubiquitinated substrates destined for degradation. Microtubule-associated protein 1 light chain 3 (LC3) or autophagy-related gene (Atg) family members then recruit these specific components to autophagosomes, resulting in fusion with lysosomes and deg [11]radation to recycle specific components [12]. It has been shown that p62 targets dengue virus (DENV) capsid proteins for autophagic degradation in an ubiquitin-dependent manner, revealing the potential role of p62 in restricting DENV replication [13]. NBR1 targets cauliflower mosaic virus (CaMV) for autophagy-dependent degradation, thereby limiting CaMV infection [14]. Furthermore, viruses exploit selective autophagy receptors to promote their replication and evade host immunity. For example, influenza A virus (IAV) degrades mitochondrial antiviral signaling protein (MAVS) via the NBR1-mediated selective autophagy pathway [15] whereas porcine reproductive and respiratory syndrome virus (PRRSV) suppresses innate immune responses through p62-mediated selective autophagic degradation of DEAD-box helicase 10 (DDX10) [16]. Collectively, these findings highlight the dual role of autophagy during viral infection, functioning either as a host defense mechanism or as a pathway hijacked by viruses to promote their survival and replication. In herpesviruses, this interaction is particularly dynamic and occurs at multiple stages of infection, including autophagy initiation, autophagosome formation, and late-stage autophagic flux. However, the regulatory strategies and biological outcomes differ substantially among herpesvirus species. A comparative overview of representative herpesviruses and their stage-specific modulation of autophagy is summarized in Figure 1.

**Figure 1.**
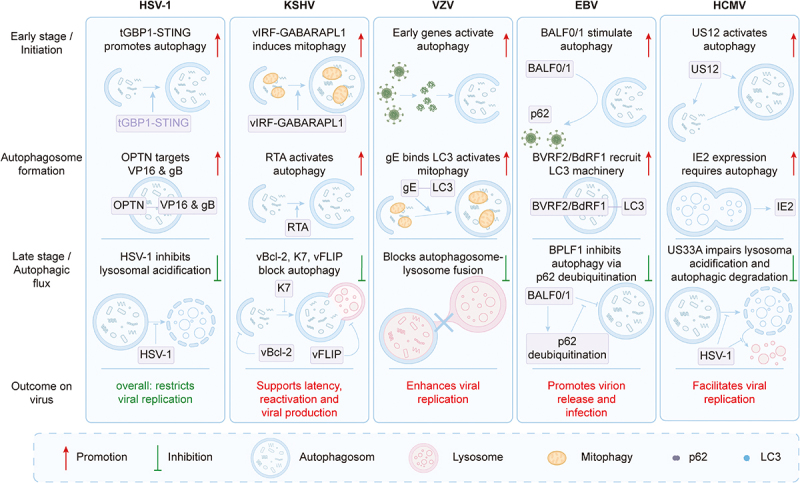
Regulation of autophagy by herpesvirus proteins at distinct stages of the autophagic pathway.

This schematic summarizes representative viral proteins from HSV-1, KSHV, VZV, EBV, and HCMV that modulate autophagy initiation, autophagosome formation, mitophagy, and late-stage autophagic flux. During the initiation stage, HSV-1 tGBP1–STING signaling promotes autophagy, KSHV vIRF – GABARAPL1 induces mitophagy, VZV early genes activate autophagy, EBV BALF0/1 stimulates autophagy, and HCMV US12 activates autophagy. During autophagosome formation, HSV-1 OPTN targets the viral proteins vPF6 and gB, KSHV RTA activates autophagy, VZV gE binds LC3 to induce mitophagy, EBV BVRF2 and BdRF1 recruit the LC3 machinery, and autophagy supports HCMV IE2 expression. At the late stage of autophagic flux, multiple viral proteins interfere with autophagosome maturation or lysosomal degradation: HSV-1 inhibits lysosomal acidification; KSHV vBcl-2, K7, and vFLIP block autophagy; VZV blocks autophagosome – lysosome fusion; EBV BPLF1 inhibits autophagy through p62 ubiquitination; and HCMV US33A impairs lysosomal acidification and autophagic degradation. These stage-specific regulatory events result in distinct virological outcomes. Autophagy generally restricts HSV-1 replication, whereas KSHV-mediated autophagy modulation supports latency, reactivation, and virion production. VZV and HCMV exploit autophagy-related processes to enhance viral replication, while EBV autophagy regulation promotes virion release and infection. Red arrows indicate activation or promotion, green inhibitory symbols indicate suppression, and the schematic icons represent autophagosomes, lysosomes, mitophagy, p62, and LC3.

In summary, the Herpesviridae family is characterized by its diversity and complex survival strategies, which are exemplified by its interactions with the autophagy pathway. On the one hand, cellular autophagy, as an integral part of the innate immune system, acts to limit viral infections by recognizing and degrading viral components. On the other hand, herpesviruses have evolved mechanisms to evade the antiviral effects of autophagy by manipulating autophagic pathways (Figure 2). This intricate interplay underscores the dynamic relationship between viruses and hosts, providing a foundation for the development of novel antiviral strategies targeting virus – autophagy interactions. Ultimately, these insights deepen our understanding of herpesvirus life cycle regulation and provide avenues for innovative therapeutic interventions.

**Figure 2.**
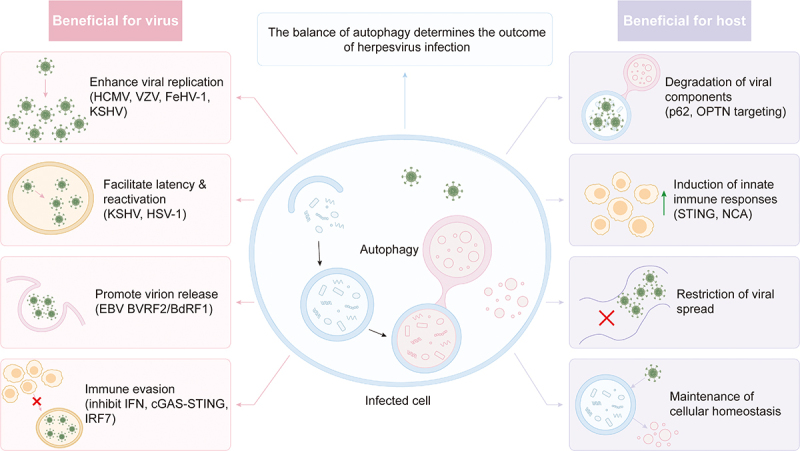
Dual roles of autophagy in herpesvirus infection. This schematic illustrates the context-dependent effects of autophagy on both viral infection and host antiviral defense. In infected cells, autophagy functions as a dynamic and bidirectional process whose overall outcome depends on the balance between virus-beneficial and host-beneficial effects. On the virus-beneficial side, herpesviruses can exploit autophagy or autophagy-related membranes to enhance viral replication, as observed for HCMV, VZV, FeHV-1, and KSHV. Autophagy may also facilitate viral latency and reactivation, particularly in KSHV and HSV-1 infection, and promote virion release through viral proteins such as EBV BVRF2 and BdRF1. In addition, several herpesviral proteins inhibit antiviral immune signaling, including IFN responses, cGAS – STING signaling, and IRF7-mediated pathways, thereby contributing to immune evasion. In contrast, autophagy can also benefit the host by restricting viral infection. Selective autophagy receptors such as p62 and OPTN can target viral components for autophagic degradation, thereby limiting viral replication. Autophagy-related pathways may also promote innate immune responses through molecules such as STING and NCA, restrict viral spread, and help maintain cellular homeostasis during infection.

## HSV-1 manipulates autophagy through viral proteins to balance immune evasion and antiviral responses

Herpes simplex virus type 1 (HSV-1), a member of the herpesvirus alpha subfamily, establishes lifelong infections in both human and animal hosts. The majority of the global population is estimated to carry HSV-1, which can cause a range of clinical manifestations, including oral, ocular, and genital infections [17]. Furthermore, HSV-1 can spread to the central nervous system, leading to severe encephalitis and potentially contributing to the development of neurodegenerative diseases [18]. The virus invades sensory nerve tissues via retrograde axonal transport, traveling along nerve endings at the site of infection and establishing latent infection in neuronal cells [19]. Autophagy, a key innate immune mechanism, is significantly activated and involved in the antiviral response of host cells during HSV-1 infection (Table 1).

**Table 1.** Representative herpesvirus proteins involved in autophagy regulation and their molecular mechanisms.

virus	viral proteins	Effect on autophagy		Mechanism	References
HSV-1		↑	tGBP1 interacts with STING		[20]
	ICP34.5	↓	Inhibits the PKR pathway		[21]
	ICP34.5	↓	Binds to Beclin1		[21]
	US11	↓	Inhibits the PKR pathway		[22]
	VP16	↑	Interacts with Hsp90		[23]
	VP16	↑	Interacts with OPTN		[24]
	ICP0	↓	Downregulates p62 and OPTN		[25]
BoHV-1		↑	Promotes p62-mediated autophagic degradation of STING		[26]
KSHV		↓	Reduces ATG5 expression		[27]
	vBcl-2	↓	Inhibits Beclin1		[4]
	K7	↓	Interacts with Rubicon		[6]
	KapB	↑	Mediates PB degradation via NDP52		[28]
	KapB	↑	Promotes Beclin1 phosphorylation		[28]
	vFLIP	↓	Binds to ATG3		[8]
	vIRF-1	↑	Interacts with ATG8 / GABARAPL1		[29]
	RTA	↑	Enhances autophagy during lytic replication		[30]
γ-HV68	vBcl-2	↓	Inhibits Beclin1-mediated autophagy		[5]
VZV	gE	↑	Co-localizes with LC3B and Rab11		[31,32]
	gE	↑	Regulates mitophagy		[33]
EBV		↑	Degradation of Hsp90		[34]
	BPLF1	↓	Deubiquitinates p62		[32]
	BVRF2	↑	Interacts with LC3B		[35]
	BdRF1	↑	Interacts with LC3B		[35]
	BALF1	↑	Combined with LC3		[3]
HCMV	IRS1	↓	Interacts with Beclin1		[36]
	TRS1	↓	Interacts with Beclin1		[36]
	US12	↑	Combined with LAMP2		[37]
	US12	↑	Interacts with LC3		[37]
	US33A	↓	Promotes KPC-mediated degradation of DMXL1		[38]
	IE2	↑	Upregulates LC3-II and ATG3		[39]
PRV	VP16	↑	Promotes p62-mediated autophagic degradation of VP16		[40]
	UL21	↑	Promotes autophagic degradation of cGAS		[41]

### HSV-1 proteins inhibit autophagy

The relationship between herpesviruses and autophagy is complex. Herpes simplex virus type 1 (HSV-1) ICP34.5 protein binds to the autophagy regulator Beclin1, thereby inhibiting autophagy, and also interacts with protein phosphatase 1 (PP1). The five proteins of HSV-1 can interact with PP1, thus facilitating the dephosphorylation of eukaryotic translation initiation factor 2 (eIF2). This interaction serves to antagonize the protein kinase R (PKR) signaling pathway, exerting an indirect regulatory effect on the occurrence and development of autophagy. When HSV-1 is deficient in ICP34.5, a reduction in neurotoxicity and viral replication has been observed in mice. These findings indicate that HSV-1-mediated inhibition of autophagy contributes to efficient viral replication and neurovirulence in vivo. Importantly, however, ICP34.5 is not the only HSV-1 protein involved in the regulation of autophagy, nor is it the sole viral factor employed to inhibit this process [21]. Compared with the ICP34.5 protein, the Us11 protein is expressed at a later stage of HSV-1 infection. The Us11 protein, in particular, has been shown to impede autophagy induction through direct inhibition of PKR activation, thus hindering the phosphorylation of eIF2 and, consequently, the activation of the PKR/eIF2 signaling pathway [22]. It has also been demonstrated that the Us11 protein encoded by HSV-1 can not only inhibit autophagy by blocking PKR but also affect TANK-binding kinase 1 (TBK1) production by interacting with tripartite motif containing 23 (TRIM23), which interacts with TBK1 and heat shock protein 90 (Hsp90) to form a functional complex involved in the regulation of autophagy-mediated antiviral defense. Specifically, the Us11 protein has been shown to bind to the ARF structural domain in TRIM23. This binding impedes TBK1 recruitment to the TRIM23 complex, disrupting the TRIM23-TBK1-Hsp90 assembly through physical exclusion of TBK1 and resulting in a marked reduction in autophagosome formation [42]. This suppression of autophagy-mediated antiviral defense may create a more favorable intracellular environment for HSV-1 replication.

The HSV-1 VP16 protein interacts with heat shock protein 90 alpha (Hsp90α), and pharmacological inhibition of Hsp90 suppresses HSV-1 infection and reduces VP16 expression. with Hsp90, and the inhibition of Hsp90α has been shown to reduce VP16 expression. These findings suggest that Hsp90α may indirectly promote the transcription of the Hsp90α gene by linking with VP16, thus effectively facilitating viral infection. Conversely, treatment of HSV-1-infected mice with Hsp90 inhibitors resulted in a substantial improvement in skin lesions, accompanied by a decrease in the expression levels of the VP16 and Hsp90α genes. These findings suggest that Hsp90α may serve as a viable therapeutic target for HSV-1 infection [23].

In addition to directly inhibiting autophagy, viruses have evolved a series of mechanisms to evade host resistance, including the regulation of autophagy through proteins that recognize autophagy cargo receptors. ICP0 is a critical early protein in HSV-1, and in the early stages of HSV-1 infection, ICP0 has been shown to regulate autophagy by downregulating the p62 and OPTN. When the expression of p62 and OPTN is reduced, the body’s antiviral response is enhanced because p62 inhibits type I interferon production by binding to stimulator of interferon genes (STING), which promotes its autophagic degradation under normal conditions. Furthermore, p62 overexpression in cells has been shown to result in a significant reduction in viral production. These observations further demonstrate that modulation of selective autophagy and STING signaling plays an important role in regulating HSV-1 replication efficiency. Moreover, counteracting the host defense response through the proteasome pathway can facilitate the virus’s evasion of host defense mechanisms and promote the completion of its life cycle [25].

### HSV-1 proteins promote autophagy

HSV-1 has been found to infect Chinese tree shrews (Tupaia) and initiate autophagy to limit viral replication. Specifically, HSV-1 infection of Tupaia leads to the recruitment of tupaia guanylate binding protein 1 (tGBP1) through interactions with STING. The interaction results in the formation of a complex that recruits SQSTM1 and LC3, thus initiating the autophagy process and degrading viral particles and thus limiting viral infection [20]. Notably, HSV-1 has also been shown to elicit an autophagy response in neurodegenerative diseases. The OPTN has been shown to target the specific proteins VP16 and glycoprotein B (gB) of HSV-1 for degradation, thus impeding viral spread within the central nervous system [24]

The degradation of the virus by autophagy receptors is not exclusive to HSV-1; it has also been observed during bovine herpesvirus 1 (BoHV-1) infection, highlighting the importance of autophagy receptors in this process of autophagy. In summary, bovine herpesvirus 1 (BoHV-1) infection promotes the expression of DNA damage-inducible transcript 3 (DDIT3), a transcription factor that plays a crucial role in regulating viral replication. DDIT3 upregulates the p62-mediated autophagy – lysosomal pathway, thereby facilitating autophagic degradation of STING and suppressing the production of interferon-β (IFN-β) and interferon-stimulated genes (ISGs). This indirect effect involves the inhibition of IFN-Is and ISGs, thus creating a favorable environment for the replication of BoHV-1 [26].

Recent studies have demonstrated that HSV-1 can serve as a model virus to determine the antiviral role of STING in noncanonical autophagy (NCA). Furthermore, STING1 activation has been shown to induce not only classic autophagy but also NCA. Additionally, NCA is not dependent on Unc-51, such as autophagy activating kinase 1 (ULK1) or Beclin1 complexes, and can form MAP1LC3/LC3-positive structures. Furthermore, this study revealed that STING1-induced NCA can effectively inhibit HSV-1. In addition, the LC3-positive structures of STING1-induced NCA can fuse with lysosomes, and HSV-1 degradation can be resumed by inhibiting lysosomal function. This indicates that the elimination of DNA viruses by NCA still requires the lysosomal pathway. Consequently, STING1-induced NCA is implicated in an effective pathway for cellular resistance to HSV-1 infection [43]. Taken together, these findings demonstrate that autophagy exerts dual roles during HSV-1 infection by either restricting viral replication through antiviral degradation pathways or being subverted by viral proteins to facilitate productive infection and immune evasion.

## Viral protein-mediated modulation of autophagy in KSHV infection: coordination of latency, lytic replication, and immune evasion

Kaposi’s sarcoma herpesvirus (KSHV) is transmitted through saliva and replicates in oropharyngeal cells [44]. For the majority of its life cycle, KSHV exists in a latent form. Patients with human immunodeficiency virus (HIV) are at high risk for KSHV infection and develop a variety of KSHV-associated malignancies [45]. The precise mechanisms by which KSHV evades the immune defenses of the host to complete its replication cycle have not been fully elucidated; however, mounting evidence points to intricate interactions between KSHV and autophagy, offering novel insights into its evasion strategies (Table 1).

### KSHV proteins inhibit autophagy

As Beclin-1 has been demonstrated to be targeted and inhibited by the cellular Bcl-2 protein, KSHV has also evolved to resist autophagy and encodes a homologue of the Bcl-2 protein (vBcl-2). vBcl-2 is a protein encoded by open reading frame 16 (ORF16) that shares sequence and functional homology with members of the Bcl-2 family [4]. vBcl-2 protects a wide range of cells from apoptosis and negatively regulates autophagy. Furthermore, the relatively low level of vBcl-2 protein expression during lysis also reduces the efficiency of KSHV in lysogenic gene expression, viral DNA replication, and infectious particle production [4]. In addition to KSHV, gamma-Herpesvirus 68 (gamma-HV68) also encodes vBcl-2 [2–5]. vBcl-2 inhibits Beclin1-mediated autophagy by directly binding to Beclin1 and disrupting its function.2. vBcl-2 can interact with the autophagy protein Beclin1 to affect autophagy, and when the Bcl-2 homologue vBcl-2 binds to the autophagy protein Beclin1, the process of autophagy is inhibited [2]. Concurrently, vBcl-2 targets lytic activation of the virus itself, an effect that is predominantly specific to γ-herpesviruses. This specificity occurs because this type of virus has a protracted presence in the host and undergoes two discrete life cycles: latent and lytic replications. It typically remains latent for an extended period following infection and undergoes rapid expression and replication in host cells after activation by transcription activators and lytic genes. In the absence of vBcl-2, the expression of viral lytic proteins and the increase in transcription are significantly inhibited, leading to a substantial decrease in viral DNA replication and the production of infectious viral particles. This finding suggests that vBcl-2 is required for viral expression of lytic proteins and that it affects both DNA replication and the production of infectious viral particles. It is therefore postulated that the regulation of autophagy serves as a mechanism to control viral replication and infectious virus particle production. These findings suggest that vBcl-2-mediated autophagy inhibition is not merely a host defense evasion strategy, but also contributes to the establishment of a cellular environment favorable for KSHV lytic replication. Excessive autophagic degradation may eliminate viral proteins or replication intermediates, whereas selective suppression of autophagy may preserve cellular homeostasis while preventing virophagy. Therefore, KSHV appears to fine-tune autophagy to balance long-term persistence with efficient viral production.

Ras homologues enriched in brain-interacting proteins involved in the ubiquitination of autophagosomes (Rubicon) have been shown to inhibit VPS34, thus regulating autophagy. The KSHV membrane protein K7 has been shown to bind to Rubicon, facilitating its interaction with the Beclin-1 complex. This interaction, in turn, inhibits the activity of VPS34, leading to the suppression of autophagy [6]. In addition, previous studies have demonstrated that K7 binds to protein-linking integrin-associated protein and cytoskeleton 1 (PLIC1) and inhibits further ubiquitination. This, in turn, results in the inhibition of PLIC1 activity, leading to the efficient degradation of inhibitor of nuclear factor kappa B (IκB) and the p53 tumor suppressor. Consequently, this inhibits p53-mediated apoptosis and promotes viral replication and infection [27]. Calpastatin (CAST) is an endogenous inhibitor of calcium-activated protease (calpain, CAPN). KSHV affects the cellular autophagy process by decreasing CAST expression, which in turn reduces the Atg5 level. This novel mechanism of action reveals a previously unobserved method by which KSHV evades immune surveillance [46]. FLIP is a class of proteins that play important roles in apoptosis regulation. FLIP controls the balance between cell survival and death by regulating the apoptotic pathway and inhibiting autophagy by binding to Atg3. KSHV encodes a homologue of FLIP, designated vFLIP, which binds to Atg3 to regulate the modification and processing of LC3 proteins during autophagy. This regulatory process, in turn, affects autophagosome formation [8].

### KSHV proteins promote autophagy

The KapB (κB) protein of KSHV has been shown to promote inflammation by disassembling cytoplasmic ribonucleoprotein (RNP) particles from processing bodies (PBs). PBs are RNP particles that are ubiquitous and regulate the expression of numerous cytokine RNA transcripts, thus establishing PBs as critical sites of inflammatory regulation. KapB-induced inflammation is facilitated by the selective autophagy receptor calcium binding and coiled-coil domain containing protein 2 (NDP52, CALCOCO2), which activates autophagy and promotes the catabolism of PBs, consequently inducing inflammation. Furthermore, KapB has been shown to enhance the autophagic process by phosphorylating autophagy protein beclin 1, thus promoting PB breakdown [28].

Viral interferon regulatory factor 1 (vIRF-1) is encoded by KSHV, which interacts with gamma-aminobutyric acid receptor-associated protein like 1 (GABARAPL1) in the autophagy-related 8 (Atg8) protein, allowing mitochondrial autophagy to begin. The reduced production of antiviral factors stimulates favorable conditions for viral reactivation and productive replication. Furthermore, the study found that the expression of the cytosolic mitogenic protein NIP3-like protein X (NIX) enhanced the interaction of vIRF-1 with GABARAPL1. In addition, the mitochondrial autophagy receptor NIX promoted the oligomerization of vIRF-1 and its interaction with the Atg8 protein. Blocking the interaction of vIRF-1 with GABARAPL1 led to a significant decrease in HHV-8 viral particle production, suggesting that normal GABARAPL1 expression and binding to vIRF-1 are essential for viral replication [29].

RTA (ORF50) of KSHV, an immediate – early gene with E3 ubiquitin ligase activity [47], targets interferon regulatory Factor 7 (IRF7) for proteasome-mediated degradation [30,48]. The binding of RTA to IRF7 has been shown to impede IRF7-mediated interferon α (IFN-α) and interferon β (IFN-β) mRNA production while concurrently promoting the ubiquitination and proteasome-dependent degradation of IRF7^37^. This, in turn, interferes with the production of type 1 IFN and disrupts the host antiviral defense mechanism. Furthermore, RTA facilitates increased activation of autophagy during KSHV lysis and replication. In summary, RTA has been shown to activate autophagy. Furthermore, studies have demonstrated that the inhibition of autophagy decreases expression of RTA-mediated lysis genes and reduces KSHV lytic replication, suggesting a role for autophagy in KSHV lytic replication [49]. The observation that autophagy inhibition suppresses RTA-mediated lytic replication indicates that autophagy is functionally required during productive KSHV infection. Mechanistically, autophagy may provide metabolic substrates and membrane resources necessary for efficient viral replication, while simultaneously limiting antiviral innate immune signaling. Thus, KSHV appears to exploit autophagy as a proviral process during the lytic phase.

These findings indicate that KSHV may carry genes such as vBcl-2, vFLIP, and K7 through its unique evolutionary process. These genes have the potential to inhibit various steps of the autophagy pathway, which could ultimately affect the life cycle of the virus. In the future, discovering additional proteins capable of interacting with autophagy may facilitate a more comprehensive understanding of the mechanisms underlying these interactions. This, in turn, may lead to the development of more effective drugs that can inhibit virus replication through these mechanisms and targets. Collectively, current evidence suggests that KSHV does not uniformly inhibit or activate autophagy, but instead dynamically remodels distinct autophagic processes according to different stages of the viral life cycle. During latency, suppression of autophagy may protect viral components from degradation and support persistent infection. In contrast, during lytic replication, selective activation of autophagy and mitophagy appears to facilitate viral gene expression, metabolic adaptation, immune evasion, and virion production. These findings indicate that autophagy functions as a double-edged regulator in KSHV infection and highlight the sophisticated evolutionary adaptation of KSHV to exploit autophagic pathways for long-term survival and productive replication.

## Stage-specific regulation of autophagy by VZV: activation of early autophagy and blockade of autophagic flux

Varicella zoster virus (VZV) is an α-herpesvirus that infects more than 90% of the global population and leads to varicella and herpes zoster [50]. It establishes a lifelong latent infection in neurons of the trigeminal ganglion and dorsal root ganglion [51], which reactivates in approximately one-third of infected individuals later in life, resulting in herpes zoster. Clinical infection with VZV not only causes chickenpox or herpes zoster but also may lead to many complications, such as stromal keratitis and cerebrovascular stroke, and may even be life-threatening in severe cases [50]. Autophagy plays a dual regulatory role in VZV infection (Table 1), suggesting that an in-depth study of the molecular mechanism of the autophagy pathway could provide an important theoretical basis for the development of targeted and precise therapeutic strategies.

As early as 2009, Marie-Noëlle Takahashi discovered that VZV can activate autophagy and that this activation is initiated by early viral genes [52]. Johanna L. Heinz et al. reported that VZV significantly increased the conversion of LC3-I to LC3-II and that VZV could not elicit autophagy when inactivated by UV light. They further reported that the use of autophagy inducers decreased viral transcription and replication and that viral replication was enhanced when Atg were knocked down [53]. In a separate study, Erin M. Buckingham et al. reported that the use of autophagy inhibitors or the knockdown of Atg led to a significant decrease in the expression of viral glycoprotein E (gE). Furthermore, their research indicated that the VZV gE glycoprotein colocalized in cytoplasmic vesicles with LC3B and Ras-related protein (Rab11) in the brain [31]. In contrast, Soo-Jin Oh et al. reported that VZV utilizes gE to regulate mitochondrial autophagy and inhibits the STING and MAVS pathways to weaken the host innate immune response. Specifically, gE binds LC3 and promotes the formation of autophagosomes, and the induction of mitochondrial autophagy enhances viral replication [33].

In contrast, Chiharu Graybill et al. reported that VZV regulates late autophagy and VZV infection inhibits autophagosome‒lysosome fusion and subsequent degradation processes despite autophagosome generation. By regulating autophagy, VZV infection inhibited autophagosome degradation. The inhibition of autophagy has been found to increase viral titers, whereas an increase in autophagy has been found to decrease viral production. This finding suggests that VZV may inhibit autophagosomal fusion and lysosomal autophagy by blocking autophagosome‒lysosome fusion to evade host degradation and promote its own replication [10].

These findings suggest that autophagy plays a dual and stage-specific role during VZV replication. Early autophagy activation may provide membrane resources, metabolic substrates, and vesicular trafficking platforms that support viral protein synthesis, glycoprotein processing, and virion assembly. However, excessive autophagic degradation may eliminate viral proteins and progeny virions through virophagy. Therefore, VZV appears to selectively activate early stages of autophagy while impairing autophagic flux at the late stage, particularly by disrupting autophagosome maturation and degradation, thereby preserving the proviral benefits of autophagy while preventing degradation of viral components. In addition, VZV-induced mitophagy may further facilitate viral replication by suppressing mitochondria-associated innate immune signaling pathways, including STING and MAVS. Overall, the ability of VZV to differentially regulate early autophagy and late autophagic degradation highlights the complex interplay between viral survival strategies and host defense pathways. A deeper understanding of these mechanisms may provide important insights for the development of precise antiviral therapies targeting autophagy-related pathways.

## EBV-encoded proteins orchestrate autophagy to regulate viral replication, release, and epithelial infection

Epstein-Barr virus (EBV), also known as human herpesvirus 4 (HHV-4), is a member of the Gammaherpesvirinae subfamily and the first identified human oncogenic virus, which is closely associated with several epithelial and lymphoid malignancies [11,54]. EBV is transmitted primarily through saliva. However, breast milk, body fluids, and transplanted EBV-positive organs can also serve as vectors for the transmission of the virus from one host to another [55]. The latent nature of EBV, which is capable of remaining in human B cells for extended periods, and its subsequent activation and proliferation during periods of immunocompromise suggest the presence of a mechanism that enables EBV to evade the host’s cellular immunity [56]. A close examination of the autophagy mechanism revealed that EBV employs a sophisticated immune evasion strategy, effectively countering the surveillance and elimination efforts of the innate immune system (Table 1).

Two proteins, designated BALF0 and BALF1, have been identified as viral homologues of the vBcl-2. These proteins play pivotal roles in the initial stages of the viral life cycle, particularly during the lysis phase. The expression of BALF1 has been shown to induce p62 accumulation. This observation indicates that p62 evades the process of autophagic degradation in the presence of BALF1. Furthermore, BALF1 accumulates in LC3-positive vesicles, and studies have identified a potential region, the LIR, within BALF1 that interacts with LC3, facilitating its binding to the autophagy-associated protein LC3 and thus stimulating autophagy. These findings suggest a potential role for BALF0/1 in the mechanism by which EBV utilizes autophagy to promote viral particle formation [3].

BPLF1, a deubiquitinating protein of EBV, interacts with p62, leading to p62 deubiquitination. This, in turn, affects the ability of p62 to recruit LC3 and the formation of autophagic vesicles, thus inhibiting autophagy. Furthermore, by modulating p62 deubiquitination, this mechanism may facilitate the evasion of the host autophagy defense mechanism by EBV during infection, thus promoting viral infection, replication, and the production of infectious viral particles [32].

The capsid scaffolding proteins BVRF2 and BdRF1 promote the release of viral particles by interacting with the LC3B membrane and facilitating the release of viral particles. This finding suggests that EBV directly interacts with the membranes of autophagy vacuoles through these proteins and utilizes the conjugation complex of LC3B (Atg5-Atg12-Atg16L1) to promote the release of viral envelopes, allowing mature viral particles to enter the extracellular milieu and thus continue to infect other cells. In the absence of BVRF2/BdRF1 or Atg5-Atg12-Atg16L1, the release of the viral envelope is impaired. It has been demonstrated that BVRF2 and BdRF1, in conjunction with Atg5-Atg12-Atg16L1, orchestrate the release of the viral envelope of EBV in a living organism [35].

Furthermore, EBV infection in nasopharyngeal epithelial cells can be modulated by autophagy. The E3 ubiquitin ligase TRIM26 has been shown to induce the degradation of ubiquitinated heat shock protein 90 beta (Hsp90β) by specifically targeting it via the proteasomal pathway. The degradation of HSP90β significantly affects the epithelial receptor ephrin type-A receptor 2 (EphA2), which plays a crucial role in the entry and infection of EBV into epithelial cells. EphA2 has been found to bind to the virus, thus facilitating the process of viral infection. The indirect reduction in the expression of the EphA2 receptor on the cell surface is achieved by decreasing the level of HSP90β, consequently hindering effective invasion of EBV and reducing the efficiency of viral infection. Notably, EBV infection itself upregulates HSP90β expression, and increased HSP90β levels promote EphA2 expression, creating a positive feedback mechanism. In summary, these findings reveal a novel feedback mechanism for EBV infection of epithelial cells and underscore the potential of TRIM26 as a therapeutic target for anti-EBV infection [34].

Taken together, current evidence indicates that EBV dynamically exploits distinct stages of the autophagy pathway to support viral replication, virion maturation, release, and epithelial infection. Autophagy-associated membranes and LC3 conjugation machinery may provide structural platforms for viral assembly and envelope trafficking, thereby facilitating the efficient production and extracellular release of infectious virions. At the same time, selective modulation of p62-dependent autophagy may help EBV evade autophagic degradation and maintain intracellular viral components. Furthermore, EBV-induced regulation of host factors associated with epithelial entry suggests that autophagy-related pathways contribute not only to intracellular replication but also to viral dissemination and host cell tropism. These observations highlight the multifaceted role of autophagy in coordinating EBV persistence, productive infection, and immune evasion.

## Viral protein-mediated modulation of autophagy and lysosomal function in HCMV infection

Human cytomegalovirus (HCMV) is a member of the β-herpesvirus subfamily and is associated with various congenital defects, including infections, hearing impairment, and other birth defects in infants [57]. The virus can be transmitted from mother to child during pregnancy or childbirth, as well as through saliva, breast milk, urine, vaginal secretions, and semen. Most HCMV infections remain latent, meaning that they do not actively replicate but can become active and cause disease when the immune system is weak [58]. The relationship between HCMV and autophagy is highly important in virological studies, especially in understanding how HCMV utilizes or modulates the autophagic machinery of host cells to facilitate their replication, latency, and reactivation. After infecting host cells, HCMV can interact with the autophagic pathway through a variety of mechanisms (Table 1), demonstrating complex viral escape mechanisms from the host’s innate immunity and modulation of the viral life cycle strategy.

HCMV has been shown to regulate autophagy throughout the viral cycle. The virus can initially activate the formation of autophagosomes and subsequently impede the fusion of these vesicles with lysosomes, thus suppressing lysosomal degradation [59]. Earlier studies revealed that at least two viral proteins, IRS1 and TRS1, participate in the inhibition of autophagy. These two highly homologous proteins, encoded by HCMV, bind to Beclin1 to inhibit autophagy. Autophagy activation increases the infectivity of HCMV, whereas its inhibition reduces viral production. Moreover, autophagy suppression severely impairs viral DNA replication, while its induction does not enhance replication. Conversely, the inhibition of autophagy appears to exert its effect during the early stages of viral replication [39].

The HCMV protein US12 directly binds lysosomal membrane protein 2 (LAMP2) and LC3, and it strongly colocalizes with LC3. Even under nutrient-sufficient conditions, cells overexpressing US12 can activate autophagy and autophagosome formation by promoting ULK1 phosphorylation and accelerating LC3-II conversion. Furthermore, US12 has been shown to interact with p62, thus protecting it from autophagosomal degradation, resulting in an aberrant accumulation of p62 during autophagy activation. These findings suggest that p62 is resistant to autophagy-mediated degradation. A more comprehensive understanding of the molecular mechanisms underlying US12-mediated autophagy regulation may prove beneficial for the development of potential targeted antiviral therapies against HCMV [37].

Autophagy of the HCMV protein IE2 enhances the expression of IE2, autophagy enhances viral replication, and the inhibition of autophagy reduces viral particle production [39]. IE2 plays a pivotal role in autophagy induction during the early stage of HCMV infection. Furthermore, studies have shown that autophagy inhibitors and small interfering RNA targeting Atg3 (siAtg3) significantly inhibit IE2 expression, whereas autophagy activators promote IE2 expression, which provides a mechanism by which autophagy facilitates HCMV replication during the early stage of infection. However, the precise mechanisms by which autophagy controls IE2 expression remain to be elucidated and require further investigation [60].

HCMV-induced degradation of dmx-like protein-1 (DMXL1) has been shown to inhibit lysosomal acidification, the degradation of autophagic cargo, and the formation of virion assembly compartments (VACs). This process involves the acidification of vesicles by DMXL1, which interacts with V-ATPase23. The US33A protein, in turn, has been shown to inhibit the expression of IE2 through Kip1 ubiquitination-promoting (KPC) to degrade DMXL1, thus inhibiting lysosomal acidification, the degradation of autophagy cargo, and the formation of vACs, as well as reducing viral replication. The net effect of US33A is a delay in the formation of vACs and a reduction in viral replication [38].

Taken together, these observations indicate that autophagy exerts dual and stage-dependent effects during HCMV infection. Early autophagy activation appears to facilitate viral replication by promoting IE2 expression, membrane remodeling, and virion assembly. In contrast, excessive autophagic degradation and lysosomal activity may negatively affect viral persistence by promoting degradation of viral components and limiting virion assembly compartment formation. Therefore, HCMV appears to dynamically modulate distinct stages of autophagy and lysosomal function to preserve the proviral benefits of autophagy while restricting antiviral degradation pathways.

## PRV exploits autophagy to regulate viral protein turnover and suppress cGAS-STING – mediated antiviral responses

The pseudorabies virus (PRV) is the causative agent of pseudorabies, an acute viral infection [61]. Pigs are particularly vulnerable to the infection, which is transmitted primarily through the mouth and nose. Following initial infection of epithelial cells in the upper respiratory tract, the virus can spread to the nervous system and internal organs, leading to complications such as abortion, stillbirth, mummified fetuses, and respiratory disorders in gestating sows. Neonates infected with PRV often exhibit neurological symptoms, and the mortality rate can reach 100% [62]. This has resulted in significant economic losses for the animal husbandry industry. Furthermore, during the evolution of PRV, immune evasion strategies have emerged, impeding complete clearance by the host’s immune system. This process is believed to involve the regulation of the host’s autophagy mechanism [63]. PRV has evolved the ability to utilize or inhibit autophagy as part of its immune evasion strategy, further complicating defense and control measures (Table 1). Ubiquitin-specific peptidase 14 (USP14), a member of the deubiquitinating enzyme family, is responsible for removing ubiquitin from proteins through a process known as deubiquitination [64]. The ubiquitinated VP16 protein has been shown to bind to the autophagy receptor p62, a process that can promote the degradation of VP16 through the autophagy pathway. USP14 plays a pivotal role in this process, as it first interacts with VP16, and inhibition of USP14 triggers endoplasmic reticulum stress and autophagy. Then, p62 binds to ubiquitinated VP16 to contribute to VP16 degradation via selective autophagy. Inhibition of USP14 indirectly enhances autophagy through endoplasmic reticulum stress, thereby increasing the degradation efficiency of VP16 and affecting viral replication. Consistently, USP14 inhibition reduces pseudorabies virus (PRV) replication, whereas reconstitution of USP14 in USP14-deficient cells restores viral replication.VP16 has been demonstrated to regulate the transcription of direct early genes and to participate in the process of nucleocapsid assembly and maturation in the cytoplasm, a process that is essential for viral replication [40].

Cyclic GMP-AMP synthase (cGAS) functions as a DNA sensor within the cytoplasm that is activated when the cGAS pathway binds to viral or cell-associated DNA within the cytoplasm. This activation results in the generation of cyclic GMP-AMP (cGAMP), which specifically binds to and activates STING. This, in turn, leads to the recruitment and activation of TBK1 and the IκB kinase complex (IKK), resulting in the phosphorylation of interferon regulatory factor 3 (IRF3) and nuclear factor kappa-light-chain-enhancer of activated B cells (NF-κB). The NF-κB pathway plays a pivotal role in the induction of many genes, including interferons and interleukins [65,66].

UL21, a conserved periplasmic protein of α-herpesviruses, is important for viral replication and pathogenesis [67]. In PRV, the UL21 gene is the primary neurovirulence determinant, and recent studies have shown that the PRV tegument protein UL21 impedes type I interferon signaling by inducing cGAS degradation via the autophagy lysosomal pathway. The UL21 protein interacts with the selective autophagy receptor toll-interacting protein (TOLLIP) to recognize ubiquitinated CGAS and ultimately fuses with lysosomes to degrade CGAS, leading to type I interferon inhibition and increased viral replication efficiency [41].

## Dose- and time-dependent autophagy induction promotes FeHV-1 replication via SQSTM1/p62-associated mechanisms

Feline herpesvirus 1 (FeHV-1), a member of the Herpesviridae family, is distributed worldwide and causes feline viral rhinotracheitis. FeHV-1 induces autophagy in a viral dose- and time-dependent manner. FeHV-1 infection has been shown to increase autophagy in feline renal cells in a viral dose- and time-dependent manner. The use of autophagy inhibitors has been found to attenuate viral titers, whereas the use of inducers has been found to increase the viral load. These findings suggest that autophagy facilitates FeHV-1 replication. These findings, which revealed that the inhibition of FeHV-1 replication could be achieved through the inhibition of autophagy, as evidenced by the knockdown of SQSTM1 or the expression of p62, provide novel insights into the potential development of therapeutic strategies. This understanding is predicated on a comprehensive examination of the biology and pathogenesis of FeHV-1 infection [68].

Taken together, these findings indicate that autophagy plays a proviral role during both PRV and FeHV-1 infection by regulating viral protein turnover, suppressing antiviral innate immune signaling, and facilitating productive replication. Selective autophagy pathways involving p62/SQSTM1 appear to contribute to the dynamic control of viral protein stability and intracellular viral homeostasis, thereby influencing viral replication efficiency. In PRV infection, autophagy-mediated degradation of cGAS through the UL21–TOLLIP axis suppresses STING-dependent antiviral responses and creates a cellular environment favorable for viral replication. Meanwhile, FeHV-1-induced autophagy may provide metabolic support and intracellular trafficking platforms that facilitate viral propagation. These observations suggest that herpesviruses can exploit distinct autophagy-associated mechanisms not only to evade host immunity but also to optimize productive infection and viral dissemination.

## MCMV and autophagy: revealing the immune evasion mechanism of murine cytomegalovirus and its regulation of host autophagy

Murine cytomegalovirus (MCMV) is classified within the genus cytomegalovirus (CMV) in the subfamily β-herpesvirus of the Herpesviridae family. Given the numerous similarities between MCMV and HCMV infections, infected mouse models have been extensively used to investigate the pathogenesis of acute, latent, and recurrent viral infections [69].

The M45 protein of MCMV contains a specific sequence termed the induced protein aggregation motif (IPAM). It induces NF-κB essential modulator (NEMO) and receptor-interacting serine/threonine-protein kinase 1 (RIPK1), which are pivotal regulators of NF-κB, to form polymers within cells. The M45 protein of MCMV can induce the polymerization of NEMO and RIPK1 within cells. This process involves the recruitment of the autophagy-associated protein VPS26B and the LC3-interacting junction protein TBC1D5, which are crucial for the degradation of these proteins by selective autophagy. This process serves to attenuate the mechanism that relies on NF-κB activation and programmed death to clear the viral infection, thus inhibiting the innate antiviral response of the host. In addition, the ICP6 protein of HSV-1 was able to induce the polymerization and autophagic degradation of RIPK1. This finding suggests that this immune escape strategy may have broad applications for the herpesvirus family [7].

## Perspective

Herpesviruses have been shown to interact with the cellular autophagy process through sophisticated and complex mechanisms, thus modulating their life cycle and influencing the host’s immune response. Several herpesviruses, including KSHV, HCMV, FHV-1, PRV, and others, have been shown to modulate autophagy through diverse mechanisms to facilitate their replication and transmission. However, studies on the interaction between herpesviruses (e.g. PRV, FHV, BoHV-1 and MDV) and autophagy remain limited. In the future, a more comprehensive study of human herpesviruses could serve as a framework for the systematic analysis of the autophagy regulation strategy employed by animal herpesviruses in host cells. This, in turn, can facilitate the development of precise prevention and control measures for animal diseases. This will not only contribute to the healthy and sustainable development of the animal husbandry industry but also play an important role in the global animal disease prevention and control system, contributing to scientific knowledge for human food security and public health safety.

**Figure 3.**
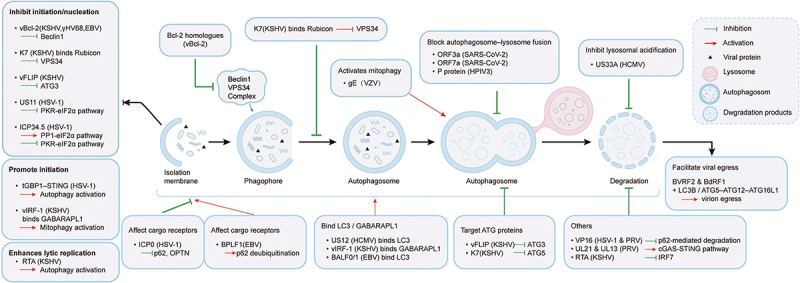
Stage-specific regulation of the autophagy pathway by herpesvirus proteins. During the initiation/nucleation stage, several herpesvirus proteins suppress autophagy by targeting the Beclin 1–VPS34 complex or upstream autophagy signaling. For example, vBcl-2 homologues encoded by KSHV, HV-68, and EBV interact with Beclin 1 to inhibit autophagy initiation, whereas KSHV K7 binds Rubicon and interferes with VPS34-dependent autophagy regulation. KSHV vFLIP can inhibit autophagy through ATG3, and HSV-1 US11 and ICP34.5 suppress autophagy-related antiviral signaling through the PKR – eIf2α pathway. In contrast, other herpesvirus proteins promote autophagy or selective autophagy. HSV-1 tGBP1–STING signaling activates autophagy, KSHV vIRF-1 interacts with GABARAPL1 to induce mitophagy, and KSHV RTA promotes autophagy activation during lytic replication. At the phagophore and autophagosome formation stages, viral proteins can directly engage LC3/GABARAP family proteins or ATG machinery. HCMV US12 binds LC3, EBV BALF0/1 binds LC3, KSHV proteins can interact with GABARAPL1, and VZV gE activates mitophagy. In addition, EBV BVRF2 and BdRF1 recruit LC3 and the ATG5–ATG12–ATG16L1 complex to facilitate virion egress. At the cargo recognition stage, herpesviruses also regulate selective autophagy receptors. HSV-1 ICP0 affects the antiviral cargo receptors p62 and OPTN, whereas EBV BPLF1 modulates p62 through deubiquitination, thereby altering autophagic targeting of viral components. During the late stage of autophagy, viral proteins interfere with autophagosome maturation and lysosomal degradation. HCMV US33A inhibits lysosomal acidification, thereby impairing autophagic degradation.

KSHV has been shown to inhibit autophagy through the action of the proteins vBcl-2 and K7 and other mechanisms, thus affecting the production and life cycle of viral particles. HCMV employs a multifaceted regulatory mechanism that involves the activation and inhibition of autophagy at various stages of replication by using proteins such as US12 to influence the formation and function of autophagosomes. Additionally, it controls the process of lysosomal acidification and the degradation of autophagy cargo through the action of DMXL1 protein assembly. FeHV-1 replication is positively regulated by autophagy, and autophagy inhibitors reduce its infectivity. PRV regulates the autophagic degradation of CGAS through the UL21 protein to inhibit type I interferon production, whereas USP14 activity affects the autophagic degradation of the VP16 protein, which in turn regulates viral replication. These viruses also indirectly affect interferon production by manipulating molecules such as cGAS and PRDX1, further regulating autophagy and immune responses. Taken together, these findings highlight the diversity and stage-specificity of herpesvirus-mediated autophagy regulation, involving both conserved and virus-specific molecular strategies (Figure 3).

This study revealed that herpesviruses use autophagy to escape host immune defense, suggesting that the autophagy pathway is a potential target for designing antiviral therapies. Future studies must explore the specific molecular mechanisms of virus‒autophagy interactions to develop effective interventions against herpesvirus infections. Additionally, understanding how cells counteract the manipulation of autophagy by viruses is crucial. The intricate balance between autophagy inhibition and activation in virus-infected cells, along with its repercussions on antiviral immunity, poses both challenges and opportunities for future research endeavors. Given the diverse roles of autophagy during herpesvirus infection, pharmacological modulation of autophagy has emerged as a potential antiviral strategy. Autophagy inhibitors may suppress viral replication in viruses that depend on autophagic machinery for virion assembly, intracellular trafficking, or immune evasion, whereas autophagy inducers may enhance the degradation of viral components through virophagy and strengthen antiviral innate immune responses. However, because herpesviruses dynamically regulate different stages of autophagy during latent and lytic infection, excessive activation or inhibition of autophagy may produce distinct effects depending on the viral species, stage of infection, and cellular context. Therefore, a deeper understanding of the stage-specific and virus-specific functions of autophagy will be essential for the development of precise therapeutic strategies targeting autophagy pathways in herpesvirus-associated diseases.

## Data Availability

Data sharing is not applicable to this article as no new data were created or analyzed in this study.

## Abbreviations

HSV-1/2: human herpes simplex virus-1/2
MDV: chicken Marek’s disease virus
VZV: varicella-zoster virus
PRV: pseudorabies virus
FHV: feline herpesvirus
BoHV-1: bovine herpesvirus type 1
EBV: epstein-barr virus
KSHV: kaposi’s sarcoma-associated herpesvirus
